# The original polyethylene microplastics inhibit the growth of sweet potatoes and increase the safety risk of cadmium

**DOI:** 10.3389/fpls.2023.1138281

**Published:** 2023-03-07

**Authors:** Liang Shi, Zanming Chen, Yanan Hou, Jianmin Li, Zhenguo Shen, Yahua Chen

**Affiliations:** ^1^ College of Life Sciences, Nanjing Agricultural University, Nanjing, China; ^2^ The Collaborated Lab. of Plant Molecular Ecology (between the College of Life Sciences of Nanjing Agricultural University and the Asian Natural Environmental Science Center of the University of Tokyo), Nanjing Agricultural University, Nanjing, China; ^3^ National Joint Local Engineering Research Center for Rural Land Resources Use and Consolidation, Nanjing Agricultural University, Nanjing, China

**Keywords:** cadmium, polyethylene microplastics, root cortex, sweet potato, weathered

## Abstract

Microplastics (MPs) and heavy metals (HMs) co-exist in sweet potato fields of China. As the main component of agricultural field mulch and one of the most polluting and harmful HMs, the effects of polyethylene microplastics (PE MPs) and cadmium (Cd) on sweet potato and soil environment are remains unclear. Here, pot and hydroponic experiments are used to explore the effects of original and weathered PE MPs on growth and Cd uptake of sweet potatoes. The results of pot experiments reveal that compared with the control (0%), 5% of weathered PE MPs can significantly increase soil electrical conductivity (EC); both 5% of the original PE MPs and weathered PE MPs can significantly reduce the concentration of Olsen phosphorus (P) and Olsen potassium (K) in soil, inhibit plant growth, but significantly increase Cd accumulation and glutathione (GSH) level in tissues of sweet potatoes, and also induce membrane lipid peroxidation. In addition, compared to 5% weathered PE MPs, 5% original PE MPs significantly reduce soil EC, growth and peroxidase level of sweet potatoes, but significantly increase Cd concentration in leaves and stems. The results of hydroponic experiment show that original PE MPs significantly increase the P, K, and Cd adsorption compared with weathered PE MPs, and Cd increases the original PE MPs accumulation in the root cortex but decrease PE MPs accumulation in shoots. To sum up, our study investigates the differences and reasons of the effects of original and weathered PE MPs on growth and Cd absorption of sweet potatoes.

## Graphical Abstract

OPE: Original polyethylene (PE) microplastic; WPE: Weathered PE microplastic; K: Potassium; P: Phosphorus; Cd: Cadmium; EC: Electrical conductivity. **↑**: The values in original or weathered PE treatment are significant higher than that of control (0 % PE). **↓**: The values in original or weathered PE treatment are significant lower than that of control.
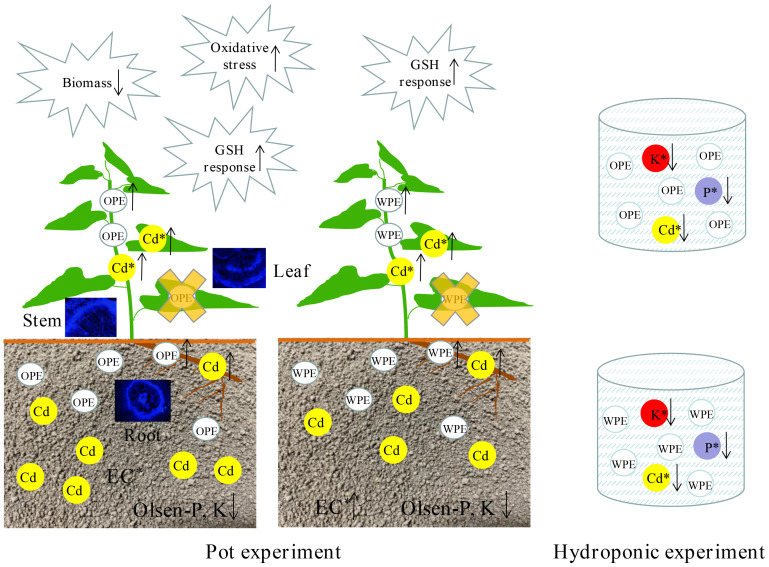

## 1 Introduction

The pollution of microplastics (MPs) in terrestrial environments has received increasing attention worldwide (Rillig et al., 2019). Being small in size with surface area, some MPs have negative effects on germination, shoot length and biomass of plants, reducing earthworm biomass and changing soil physical and chemical properties, such as by causing soil moisture loss and reducing soil porosity (Boots et al., 2019; Mbachu et al., 2021). In addition, MPs can be weathered in nature under the influence of several factors. During the weathering process, the particles are decreasing in size, surfaces are gradually becoming rougher, and various function groups are produced (Jahnke et al., 2017; Ding et al., 2020). As little is currently known regarding the changes in toxicity of MPs in soil over time, it is important to explore the impact of weathered MPs on plants and also the soil environment.

Cadmium (Cd) is one of the most toxic heavy metals (HMs) and it has been a major contaminant in agricultural soils. In China, approximately 1.3 ×10^4^ ha of cultivated soil is contaminated by Cd and 5.0×10^4^ t of Cd-contaminated rice is produced every year (Zou et al., 2021). Cd could cause various health hazards, such as nephrotoxicity, osteoporosis, neurotoxicity, carcinogenesis, genotoxicity, and teratogenicity. For non-smokers, 90% of Cd exposure occurs *via* dietary intake (Tóth et al., 2016). In plants, Cd toxicity inhibit the carbon fixation and photosynthetic activity. Cd in soil can also lead to osmotic stress by reducing the relative water content, stomatal conductance, and transpiration of leaves. Over-production of reactive oxygen species (ROS) can be induced by Cd and cause damage to organelles. Cd interferes with the transportation of mineral elements (P, Mg, K, Mn) (Nazar et al., 2012; Haider et al., 2021). Therefore, the problem of food safety risks caused by the accumulation of Cd in crop cannot be ignored.

MPs can also adsorb several pollutants (Jacky et al., 2021), and serve as a vectors of these pollutants to organisms (Barnes et al., 2009; Andrew et al., 2020; Merga et al., 2020; Aghilinasrollahabadi et al., 2021). Considerable efforts have been devoted to investigate the interaction between metal and MPs (Oz et al., 2019). The types of metallic elements that can be adsorbed by MPs include HMs, such as Cd, as well as some beneficial elements, such as Zinc (Zn), allowing improved bioavailability of these beneficial elements to plants (Hodson et al., 2017). For example, the addition of polyester microfibers did not significantly change the level of Cd in leaves of lettuce (Zeb et al., 2022). Wang et al. (2020) found that polylactic acid caused higher Cd bioavailability than polyethylene microplastics (PE MPs), but no alterations in plant Cd content. However, in general, the adsorption of HMs to MPs requires continuous attention to prevent the accumulation of MPs, and harm from other pollutants can be further aggravated by adsorption to MPs (Aghilinasrollahabadi et al., 2021). In addition, the adsorption mechanism of different pollutants to MPs and influencing mechanism for the adsorption and desorption of HMs in agricultural soils are still unclear (Guo and Wang, 2019).

Sweet potato as an important food crop worldwide ranks third in production and fifth in calorifc contribution to human diet among all crops globally (Wang et al., 2021). China is the biggest cultivator country of sweet potato in the world, and the sweet potato production area in China about 6.6 million ha, accounted for 70% of total area under sweet potato cultivation of world. In addition, the output of sweet potato in China, about 100 million metric tons, accounted for 84.4% of total world output (Gao et al., 2000; Wang et al., 2021). Because sweet potato is also the main source of starch, it can be used as a staple food and can be an important raw material for alcohol production. Therefore, relevant food safety issues cannot be ignored (Bovell-Benjamin, 2007; Ma et al., 2012; Xie et al., 2018).

Plastic films are used in approximately 20 million hectares of farmland all over the world, and the largest proportion (~90%) are found in China (Steinmetz et al., 2016). The plastic films can be degraded or weathered into micro-sized particles after been left in fields (Astner et al., 2019). Therefore, long-term accumulation of MPs in farmland may pose a considerable threat to plant, animal growth and food security (Ramos et al., 2015). However, there are no reports about the effects of PE (the main component of the field film) MPs on sweet potato growth. In addition, Cd is one of the most seriously polluted HMs in soil of China, and nearly 33.54% and 44.65% of sites in farmland and urban soils were polluted with Cd (Yuan et al., 2021). Indeed, some edible-types sweet potato cultivars can accumulate more Cd and lead (Pb) than starch-type cultivars in flesh (Huang et al., 2020a). For instance, sweet potato with larger underground biomass shows high accumulation of Uranium (1.68-5.16 mg kg^−1^) and Cd (0.78-2.02 mg kg^−1^), and would pose a health risk if consumed (Lai et al., 2020). Therefore, in order to solve the problem that PE MPs may increase the consumption risk of Cd through sweet potato ingestion, the objectives of this study is (1) whether PE MPs can increase the Cd accumulation in sweet potato and cause more severe damage in plants; (2) the differences in the effects of original PE MPs and weathered PE MPs on sweet potato and soil properties. According to the above research objectives, the detailed research contents including: (1) the effect of PE MPs (original and weathered PE MPs) on physicochemical properties of Cd contaminated soil; (2) the effect of PE MPs (original and weathered PE MPs) on the growth and the absorption characteristics of sweet potato to Cd; and (3) reveal the effect of original PE MPs on the uptake and distribution of PE MPs in tissues of sweet potato. Therefore, (1) the micron particles of original and weathered PE MPs (the main component of plastic film in field) are used in pot and hydroponic experiments to simulate the effect of PE MP particles (which transformed from plastic film) on sweet potato growth and soil environment; (2) inductively coupled plasma-optical emission spectroscopy (ICP-OES) and cryo-scanning microscope are used to determinate Cd concentration and explore PE distribution in leaves, stems and roots of sweet potatoes respectively.

## 2 Materials and methods

### 2.1 Effect of PE MPs and Cd on physiological indexes and Cd absorption of sweet potato (pot experiment)

#### 2.1.1 Original and weathered PE MPs

In this experiment, polyethylene (PE), the main component of agricultural mulch film and also the main MPs pollutant in farmland, was selected as the basic material of the MPs. Two types of PE MPs were used: (1) PE MPs powder (fragments with an average diameter of 5 μm and purity is 99.5%); (2) monodisperse blue fluorescent PE MPs (Spherical with an average diameter of 5 μm and purity is 99.7%), which purchased from Shaanxi Xinyan Bomei Biotechnology Co., Ltd. For monodisperse blue fluorescent PE MPs, the maximum excitation wavelength is 400 nm, and the maximum emission wavelength is 450 nm.

Referring to the aging method of MPs used in our previous study (Shi et al., 2022), after oxidizing the 5 μm MPs with Folin phenol weathering agent (Folin phenol weathering agent formula: 15 mL of 200 mM FeCl_2_ solution, add 970 mL of distilled water, mix well and adjust the pH to 4.0, then add 15 mL of 30% H_2_O_2_ solution) for 7 days, distilled water were added to repeatedly rinse the MPs. After the oxidant on the surface of MPs was rinsed, the MPs were dried and stored at 60 °C for the next step.

#### 2.1.2 Experimental soil

Experimental soil was collected from a sweet potato field in Ezhou, Wuhan, China (114°3’ E, 30°34’ N). The abundance of MPs in the soil was 9,072 items/kg soil, the Cd concentration was 0.025 mg/kg, and the soil pH was 5.76. One hundred milliliter CdCl_2_ mother liquor (441 mg/L) was added to 10 kg soil by spraying to make the final concentration of Cd^2+^ in the soil is 2.7 mg/kg.

According to the method of Fei et al. (2020), a total of five treatment soils were prepared: (1) control (Cd-contaminated soil without PE MPs); (2) Cd-contaminated soil with 1% (10 g/kg) original PE MPs; (3) Cd-contaminated soil with 5% (50 g/kg) original PE MPs; (4) Cd-contaminated soil with 1% (10 g/kg) weathered PE MPs; (5) Cd-contaminated soil with 5% (50 g/kg) weathered PE MPs. After been preparation, soil was dried and wet balanced for 1 month to carry out the pot experiment.

#### 2.1.3 Sweet potato variety

The sweet potato variety (*Ipomoea batatas*) used in this experiment was ‘Fucai 18’ (purchased from the Jiangsu Academy of Agricultural Sciences, Nanjing, China). The stem tips (10 cm) of the fresh ‘Fucai 18’ were cut off, and the extra leaves were removed from the cut stem tips, leaving only 2-3 new leaves. Then, they were cultivated with distilled water, which was changed every day. After root development, the distilled water was changed to 1/4 Hoagland nutrient solution and the sweet potato was cultivated for 3 days, and finally changed to 1/2 Hoagland nutrient solution and further cultivated for 3 days. Subsequently, excess leaves were trimmed off and stem tips of the sweet potato with the same weight were selected and transferred in a pot (Diameter = 15 cm; Height = 20 cm), which filled with 500 g of soil. Three sweet potato seedlings were planted in each pot, and three repetitions were set for each treatment. The sweet potatoes were treated for 15 days.

#### 2.1.4 Determination of soil physical and chemical properties

After 15 days of sweet potato planting, the soils in the pots were mixed well and air-dried for physical and chemical properties determination. The methods to determinate pH, Electrical conductivity, available phosphorus, and available potassium were referred to Bao (2000).

#### 2.1.5 Determination of Cd in plants and soils

Referring to the method of Huang et al. (2021b), the sweet potatoes were gentle collected from the soil after been treated for 15 days. Damage to the root systems was avoided, and the soil on the surfaces of the roots was rinsed off repeatedly with distilled water, and the lower halves of the roots and stems were immersed in 20 mM EDTA-2Na solution for 30 min to remove Cd adsorbed on the surfaces of the sweet potatoes. Then, the remaining EDTA-2Na on the root surfaces was rinsed with distilled water. Finally, the roots, stems, and leaves of the samples were dried in an oven at 60°C to a constant weight.

A dried plant sample was accurately weighed at 0.2500 ± 0.0050 g and put in digestion tube, and 3 mL of nitric acid-perchloric acid (87:13, v/v) mixed acid was also added to the tube. After digestion in an electric digester, 10 mL of 2.5% nitric acid was added and then shaken for reconstitution. After being kept for overnight, the supernatant was passed through a 0.45 μm filter membrane, and the filtrate was stored at 4°C. Inductively coupled plasma-optical emission spectroscopy (ICP-OES, Optima 8000, Shanghai Perkin Elmer, Co., Ltd) was used to determine the Cd concentration in the stems, roots, and leaves of the sweet potatoes. For soil samples, 0.2000 ± 0.0050 g of the dried soil sample was accurately weighed and put in a digestion tube, and 10 mL of nitric acid-perchloric acid (4:1, v/v) mixed acid was added to the tube. After digestion in an electric digester, 10 mL of 2.5% nitric acid was added to the tube and samples were shaken for reconstitution. After overnight incubation, the supernatant solutions were passed through a 0.45 μm filter membrane, and the filtrate was stored at 4°C. ICP-OES (Optima 8000, Shanghai Perkin Elmer, Co., Ltd) was used to determine the Cd concentration in the soil.

#### 2.1.6 Biomass of ‘ Fucai 18’ sweet potato

The sweet potatoes were carefully removed from the soil to maintain the integrity of the roots after being treated for 15 days. Then, the soil on the surfaces of the roots, stems, and leaves was rinsed off with distilled water repeatedly. After being rinsed, the plant samples were dried in an oven at 60°C to a constant weight, and the dry weight of each tissue was measured and recorded by one-thousandth balance.

#### 2.1.7 Antioxidant index determination

For enzyme extracts and assays (Li et al., 2013), the fresh leaves (0.1 g) were ground in liquid nitrogen, and then suspended in 0.9 mL solution containing 10 mM phosphate buffer (pH 7.4). The homogenate was centrifuged at 4°C, 2500 rpm for 10 min and the resulting supernatant was collected for determination of the activities of superoxide dismutase (SOD, A001-1-1), peroxidase (POD, A084-3-1), and glutathione (GSH, A006-1-1) using commercial assay kits purchased from Nanjing Jiancheng Bioengineering Institute (Nanjing, China).

The activity of SOD was determined by measuring the inhibiting rate of the enzyme to O_2_^−^· produced by the xanthine morpholine with xanthine oxidase using the SOD assay kit. Each endpoint assay was detected the red substances of the reaction system by absorbance at 550 nm after 40 min of reaction time at 37°C. And one unit SOD activity was defined as the quantity of SOD required to produce 50% inhibition of reduction of nitrite in 1 mL reaction solution by measuring the change of absorbance at 550 nm.

The POD activity was measured based on the change of absorbance at 420 nm by catalyzing H_2_O_2_. One unit was defined as the amount of enzyme which was catalyzed and generated 1 µg substrate by 1.0 g fresh tissues in the reaction system at 37°C. POD activity was calculated as the formula according to POD assay kit.

The activity of GSH was measured based on the absorbance of reaction products between sulfhydryl compounds and 5,5’-Dithiobis (2-nitrobenzoic Acid) at 420 nm. GSH activity was calculated as the formula according to GSH assay kit.

Lipid peroxidation was determined as the amount of malondialdehyde (MDA) based on the spectrophotometer measurement of a red-complex produced during the reaction of thiobarbituric acid (TBA) with MDA. For MDA determination, fresh leaves (0.1 g) were ground in liquid nitrogen, and then suspended in 0.9 mL extracted solution containing 50 mM phosphate buffer (pH 7.4) and 1% polyvinylpyrrolidone (PVP). Then the homogenate was centrifuged at 4°C, 3500 rpm for 10 min to collect the supernatant, which was further processed following the instruments of assay kit (MDA, A003-1-1) purchased from Nanjing Jiancheng Bioengineering Institute (Nanjing, China). Finally, the absorbance of red TBA-MDA complex was measured using a microplate reader (SpectraMax M5, USA) at 532 nm and the MDA content was calculated according to detailed instructions of the MDA assay kit.

### 2.2 Effect of PE MPs on P, K, Cd adsorption and PE MPs distribution in sweet potato (Hydroponic experiment)

#### 2.2.1 Adsorption experiment

Referring to the concentration of Cd in experimental soil, it was necessary to add available P and K to the soil, preparing a mixed solution. CdCl_2_ was used to prepare the solution and the final Cd concentration was 2.70 mg/L. First, a 54.8 mg/L CdCl_2_ solution was prepared and 50 mL of this solution was poured out and added an appropriate amounts of KCl and NaH_2_PO_4_ were added to adjust the pH to 5.76 ± 0.52, and the above solution was diluted to 500 mL. The concentrations of P and K in the solution were kept to about the original available P (600 mg/kg) and K (400 mg/kg) concentrations in the soil, respectively.

Referring to the method of Peng et al. (2019), five treatments were applied: (1) the original mixed solution; (2) 1% (10 g/L) original PE MPs mixed solution; (3) 5% (50 g/L) original PE MPs mixed solution; (4) 1% (10 g/L) weathered PE MPs mixed solution; (5) 5% (50 g/L) weathered PE MPs mixed solution. Each treatment was set three repetitions. After the mixed solutions were shaked at 150 r/min for 7 days in a shaker at 25°C, the solution was filtered with a 0.45 μm filter membrane and the P, K, and Cd concentrations in the filtrate were determined by ICP-OES (Optima 8000, Shanghai Perkin Elmer, Co., Ltd).

#### 2.2.2 Fluorescent PE MPs distribution in sweet potato tissues

Referring to the method of Li et al. (2019), the stem tips (10 cm) of the fresh ‘Fucai 18’ were cut off, and the extra leaves were removed from the cut stem tips, leaving only 2-3 new leaves. Then, they were cultivated with distilled water, which was changed every day. After root development, the sweet potato seedlings were transferred in 198 mL of 1/4 Hoagland nutrient solution (including 5 mg/L Cd) and mixed with 2 mL of 1/4 Hoagland nutrient solution (0, 2 or 6 mg of 5 μm-PE blue fluorescent MPs suspension) for 7 days. Roots, stems, and leaves were washed repeatedly with distilled water and respectively cut and embedded in cryogen. Then, 100 μm-thick slices with cryostat were placed on a glass slide and covered with a glass lid. The excitation and emission wavelengths of the biological fluorescence microscope (Axiovert 40 CFL, Zeiss, Shanghai, China) were adjusted the to 400 and 450 nm, respectively, to observe the fluorescence in the roots, stems, and leaves. After that, the samples were stored at 4°C.

For PE MPs distribution in the root, stem, and leaf sections, the pretreating methods were the are same as those used by Li et al. (2019), and the fresh root, stem, and leaf samples were rinsed repeatedly with distilled water and frozen in liquid nitrogen. A cryo-scanning microscope (SU8010, Hitachi, Shanghai, China) was then used to observe the presence of MPs in the tissues.

### 2.3 Statistical analysis

The data were processed using Microsoft Excel 2010, and the figures were drawn by GraphPad prism software 8.0. One- and two-way ANOVA was conducted by SPSS software 21.0. The Tukey’s test was used to explain the significant differences between various treatments at *p <0.05*.

## 3 Results

### 3.1 Effect of PE MPs on the physicochemical properties of Cd-contaminated soil

**Figure 1:**
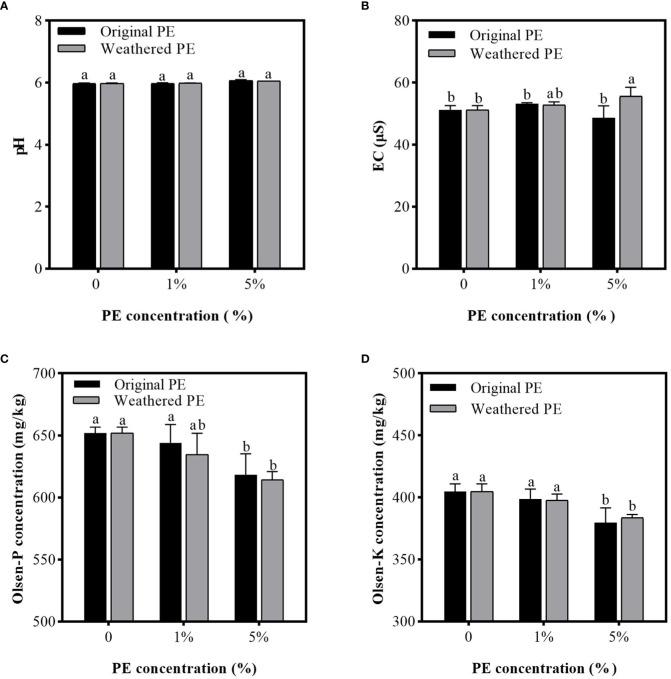
pH **(A)**, EC **(B)**, Olsen-P **(C)**, and olsen-K concentration **(D)** in PE MPs and Cd compound-contaminated soil. Soil samples are collected after sweet potatoes are treated by original or weathered PE MPs (0, 1% and 5%) for 15 days. Different letters in each figure indicate significant (p < 0.05) differences according to Tukey’s test.

**Figure 1** shows that, compared with the control group (0% PE MPs), different concentrations of original or weathered MPs only slightly affected the pH and EC of the Cd-contaminated soil, except for 5% weathered PE MPs significantly increasing EC compared with original PE MPs. As shown in **Figures 1C, D**, 1% original or weathered PE MPs did not significantly affect the concentrations of available P and K in soil compared with that of the control group (0% PE MPs), but 5% of original or weathered PE MPs significantly decreased available P and K. Compared with the control group, 5% of original PE MPs reduced the concentration of available P and K by 5.14% and 6.17%, respectively, and 5% of weathered PE MPs reduced the concentration of available P and K by 5.76% and 5.35%, respectively.

### 3.2 Effect of PE MPs on Cd accumulation in plants

**Figure 2:**
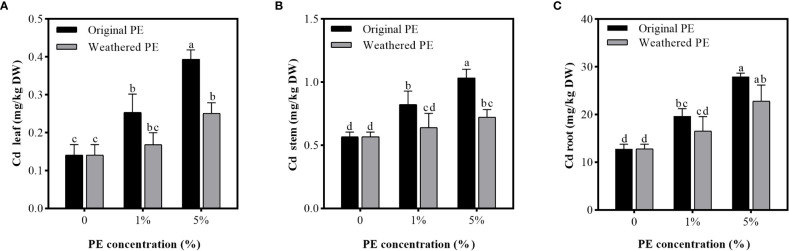
Cd concentration in the leaf **(A)**, stem **(B)**, and root **(C)** of sweet potato planting in PE MPs and Cd compound-contaminated soil. Plant samples are collected after been treated by original or weathered PE MPs (0, 1% and 5%) for 15 days. Different letters in each figure indicate significant (p < 0.05) differences according to Tukey’s test.

After the sweet potato seedlings were treated for 15 days, the Cd concentration in stems and roots exceeded the threshold of leafy vegetable safety standard (0.2 mg/kg, GB 2762-2017). In addition, compared with weathered PE MPs, original PE MPs significantly increased the Cd accumulation in stems. For leaves, the Cd concentration in control (0% PE MPs) leaves were lower than the safety standard, but 5% original or weathered PE MPs significantly increased Cd concentration in leaves, and exceeded the threshold of leafy vegetable safety standard. Furthermore, compared to the weathered PE MPs, the original PE MPs can bring higher food safety risks (**Figure 2**).

### 3.3 Effect of PE MPs on P, K, and Cd adsorption in solution

**Figure 3:**
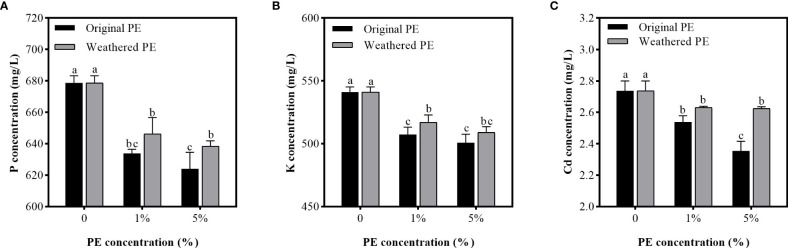
P **(A)**, K **(B)** and Cd **(C)** concentrations in PE MPs treated solutions. Solution samples are collected after been treated by original or weathered PE MPs (0, 1% and 5%) for 7 days. Different letters in each figure indicate significant (p < 0.05) differences according to Tukey’s test.

**Figure 3** shows that all PE MPs treatments significantly decreased the P, K, and Cd concentration in solution compared with control group (0% PE MPs), and with the increase in original PE MPs concentrations, the Cd adsorbtion ability of PE MPs significantly increased (**Figure 3C**). However, the adsorption of P, K, and Cd by weathered PE MPs between 1% and 5% treatments was not significantly different. Compared with weathered PE MPs, the original PE MPs at the concentration of 1% and 5% significantly reduced the K and P, Cd concentration in solution respectively. Therefore, compared with weathered PE MPs, the original PE MPs had the stronger ability to adsorb Cd.

### 3.4 Effect of PE MPs on growth and oxidative damage of sweet potato planting in Cd contaminated soil

**Figure 4:**
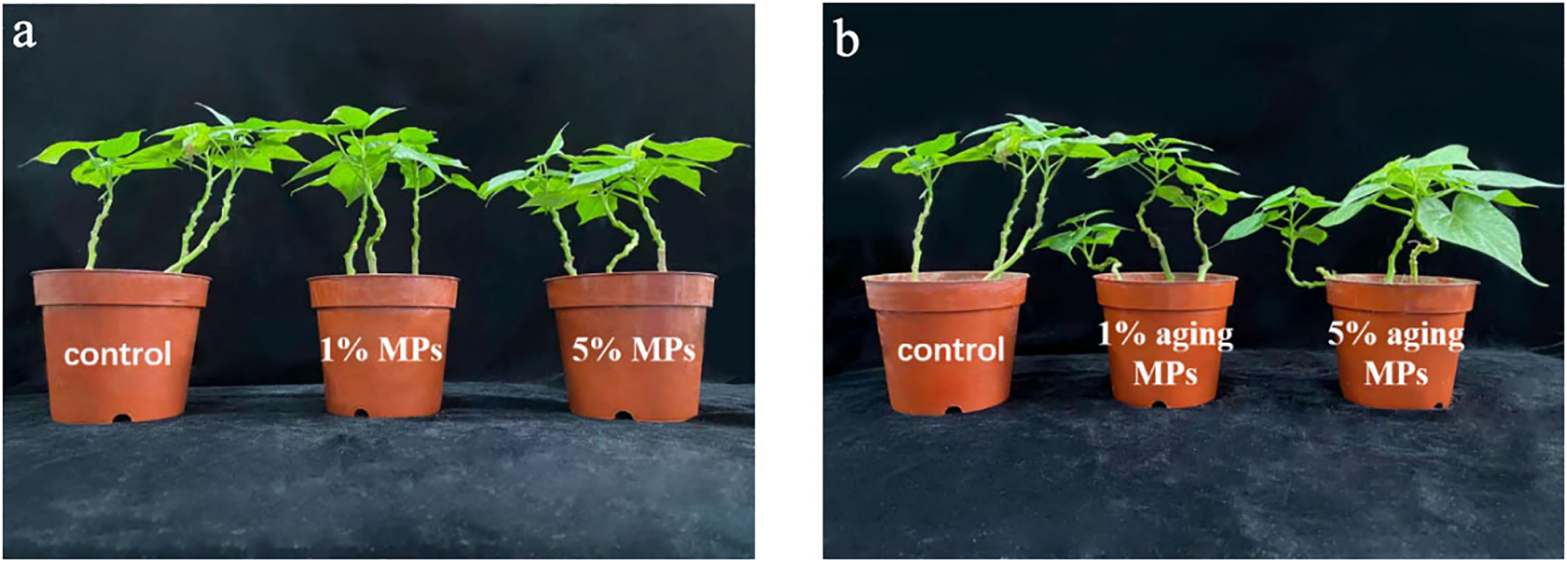
Growth of sweet potatoes planting in PE MPs and Cd compound-contaminated soil. Plant samples are collected after been treated by original **(A)** or weathered **(B)** PE MPs (0, 1% and 5%) for 15 days. Different letters in each figure indicate significant (p < 0.05) differences according to Tukey’s test.

As shown in **Figure 4** and **Table 1**, when the concentrations of original and weathered PE MPs increased, the dry weight of sweet potato decreased. Overall, 5% original PE MPs significantly reduced the dry weight of sweet potato, and 1% original PE MPs also reduced the biomass of sweet potato by 12.18% compared with control (0% PE MPs). However, 1% and 5% weathered PE MPs reduced the dry weight of sweet potato by 7.36% and 11.68%, respectively, but did not show significant differences compared with the control (0% PE MPs).

**Table 1:** Biomass of original or weathered PE treated sweet potato planting in Cd contaminated soil.

Style	PE concentration (%)	Dry weight (g)	Dry weight proportion (%)
Control	0	1.313 ± 0.04 ^a^	100.0% ^a^
Original PE	1% 5%	1.153 ± 0.05 ^ab^ 1.070 ± 0.13 ^b^	87.82% ± 0.04 ^bc^ 81.47% ± 0.10 ^c^
Weathered PE	1% 5%	1.217 ± 0.09 ^ab^ 1.160 ± 0.05 ^ab^	92.64% ± 0.07 ^ab^ 88.32% ± 0.04 ^bc^

Different letters in the same column represent significant differences.

**Figure 5:**
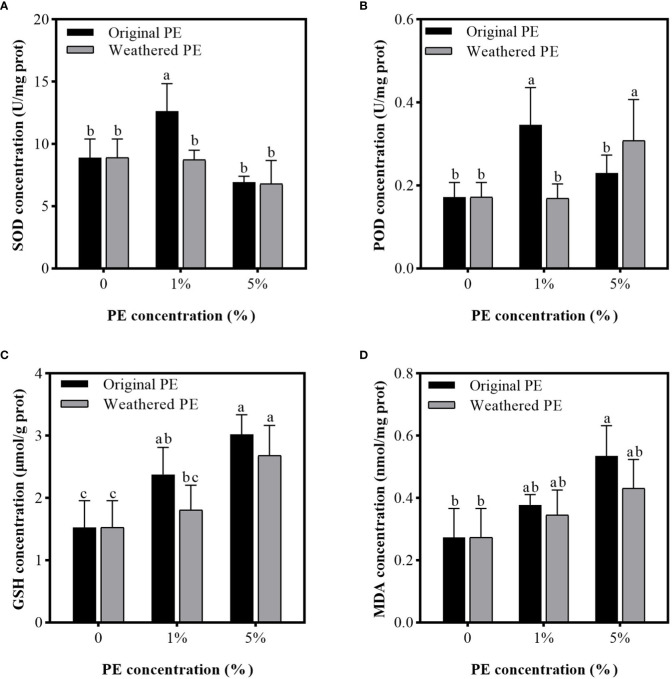
SOD **(A)**, POD **(B)**, GSH **(C)**, and MDA **(D)** concentrations in the leaves of sweet potatoes planting in PE MPs and Cd compound-contaminated soil. Plant samples are collected after been treated by original or weathered PE MPs (0, 1% and 5%) for 15 days. Different letters in each figure indicate significant (p < 0.05) differences according to Tukey’s test.

**Figure 5** shows that compared with the control group (0% PE MPs), 1% original PE MPs significantly increased the concentration of SOD, POD, and GSH enzymes, but did not significantly affect the MDA concentration. However, 5% original PE MPs significantly increased the MDA concentration. Weathered PE MPs did not significantly affect the concentrations of SOD and MDA, but 5% weathered PE MPs significantly increased the concentrations of POD and GSH compared with control group (0% PE MPs).

### 3.5 Distribution of PE MPs in the roots, stems, and leaves of sweet potato

**Figure 6:**
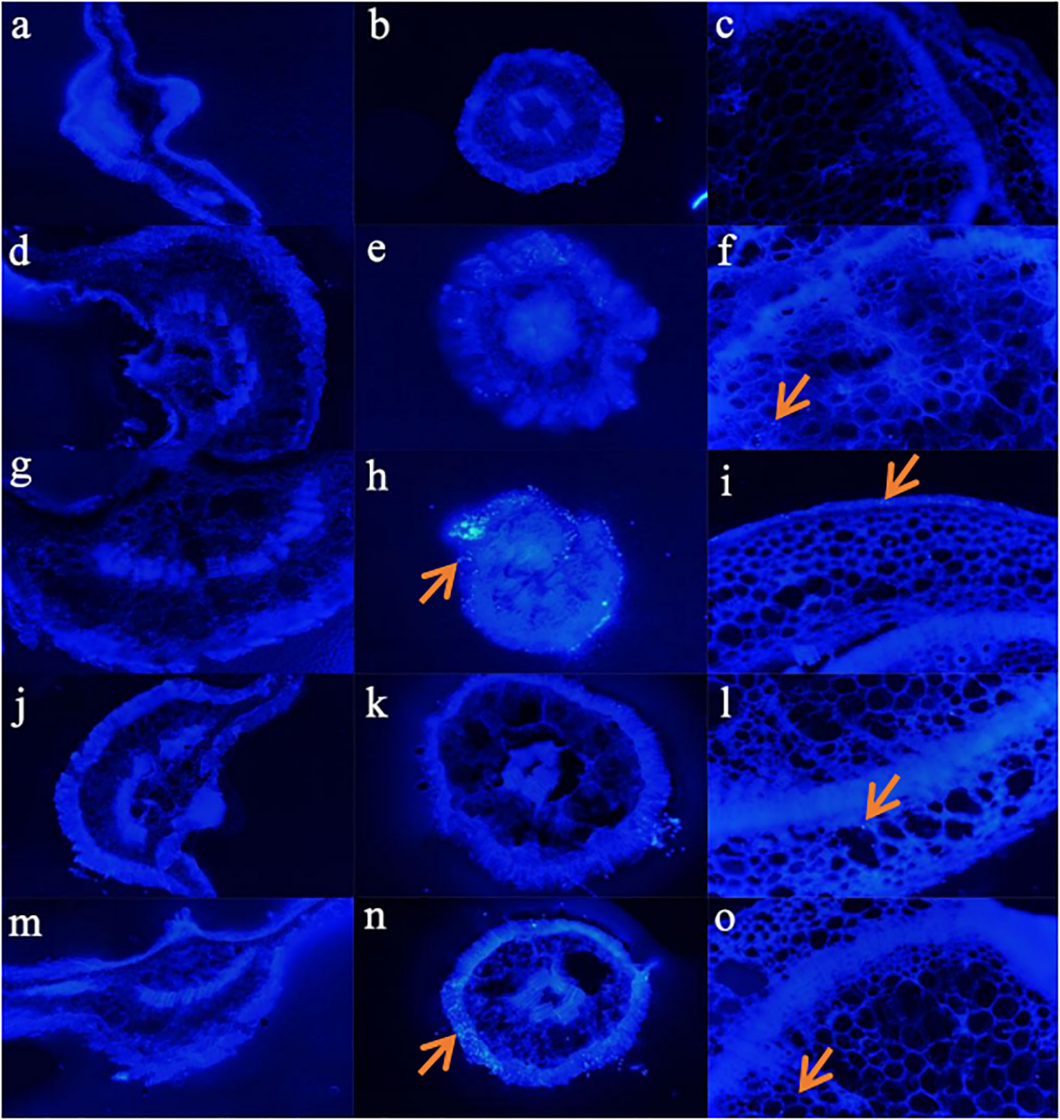
Distribution of PE fluorescent MP spheres in transverse sections of leaves **(A)**: Control, **(D)**: 1% PE MPs, **(G)**: 3% PE MPs, **(J)**: 1% PE MPs + Cd, **(M)**: 3% PE MPs + Cd), roots **(B)**: Control, **(E)**: 1% PE MPs, **(H)**: 3% PE MPs, **(K)**: 1% PE MPs + Cd, **(N)**: 3% PE MPs + Cd) and stems **(C)**: Control, **(F)**: 1% PE MPs, **(I)**: 3% PE MPs, **(L)**: 1% PE MPs + Cd, **(O)**: 3% PE MPs + Cd) of sweet potatoes. Sweet potatoes are treated with 5 μm of monodisperse blue fluorescent PE MPs and Cd for 7 days and are checked using a biological fluorescence microscope. The positions of the PE MPs are marked with red arrows.

**Figure 6** shows that there no PE MPs were found in the leaves of sweet potatoes under the five different treatments (**Figures 6A–M**). Compared with the control (0% PE MPs), higher PE MPs concentration treatment resulted in higher PE MPs accumulation in the roots, and Cd increased the PE MPs accumulation in the cortex of the root (**Figures 6B–N**). In addition, we did not find any significant differences in PE MPs accumulation in the shoots of sweet potatoes between 1% and 3% treatments (**Figures 6F–O**). However, Cd significantly reduced PE MPs accumulation in the shoots (**Figures 6F–O**).

## 4 Discussion

Despite the fact that MPs like High-density polyethylene (HDPE) have the power to decrease soil pH through the cation/proton exchange in soil (Boots et al., 2019), the soil buffering capacity and plant root exudates are able to mediate soil pH collectively. Therefore, this is the possible explanation for the no difference in soil among different treatments. Compared with 5% original PE MPs treatment, the reason for the increase in EC caused by 5% weathered PE MPs treatment may be the lower leaf transpiration rate and higher soil bulk density (Lian et al., 2021).

MPs that accumulate gradually on the ocean surface or land can be absorbed by plants (Li et al., 2019) or consumed by animals (Zhou et al., 2020a). However, the greater threat of MPs is that they can act as carriers, accumulating and combining toxic chemicals (HMs and organic pollutants) to cause a range of damage to organisms through the food chain (Angelo and Andrew, 2017; Wang et al., 2019; Huang et al., 2021a). Therefore, the co-existence of PE MPs and Cd may increase the food safety risk of sweet potato. The reason why the original PE MPs have stronger adsorption capacity for Cd compared to weathered PE MPs was that original MPs may form various groups on the surface after been weathered, and the static electricity carried by themselves were neutralized, thus, reducing the adsorption capacity (Zhou et al., 2020b). In MP and Cd combined pollution, when the Cd concentration was constant, the increasing concentration of MPs can lead to more Cd are adsorbed by MPs and greater the damage to organisms (Zhang et al., 2020). These can explain our results in soil and hydroponic adsorbtion experiments that when the concentration of PE MPs was 5%, the available P and K concentration in soil and solution were the lowest; the Cd concentration in all organs of sweet potato was highest, the biomass of the sweet potato decreased to the maximum extent; and the degree of oxidative damage was also at the highest level among all of treatments (**Table 1**, **Figures 2**–**5**). In addition, because original PE MPs can adsorb more Cd and make plants more poisonous, which lead to the higher membrane lipid peroxidation damage compared with weathered PE MPs (**Figure 5D**). However, there were no significant differences between the effects of original and weathered PE MPs on MDA concentration in plants, may be due to the stronger ability of P and K adsorption by original PE MPs compared with weathered PE MPs (**Figures 3A, B**), through which providing more nutrient for plants and increasing their tolerance to Cd.

Ali et al. (2013) found that Cd increased the level of MDA, H_2_O_2_ and Cd accumulation in the shoots of oilseed rape (*Brassica napus* L.). In addition, plants have evolved antioxidative enzyme defense system consisting of SOD, POD, catalase (CAT), ascorbate peroxidase (APX), etc (Najeeb et al., 2011), which can reduce the MDA production (Najeeb et al., 2011; Xu et al., 2016). In our study, compared with the control (0%), although the activities of SOD, POD and GSH increased significantly under the treatment of the original PE MPs at a concentration of 1%, the MDA level did not significantly reduced compared with control. It may be due to the low Cd accumulation in leaves which was induced by addition of 1% PE MPs (**Figure 2A**). However, the high Cd accumulation in leaves was induced by addition of 5% PE MPs (**Figure 2A**), and the oxidative stress in plants led to a significant increase in MDA level (**Figure 5D**). However, due to the excessive Cd accumulation, the activities of SOD and POD under 5% original PE MPs treatments were significantly inhibited compared under 1% original PE MPs group (**Figures 5A, B**). Besides, we found that POD activity in 5% weathered PE MPs treatments were significantly higher than that of 1% weathered PE MPs treatments. It was due to the lower Cd accumulation in weathered PE MPs group compared with original PE MPs group (**Figure 2**). In addition, the POD activity in the tissues under the 5% weathered PE MPs treatment was significantly higher than that of the 5% original PE MPs and control treatments (**Figure 5B**). This may be another reason why plants were subjected to lower oxidative stress under 5% weathered PE MPs treatment than under 5% original PE MPs treatment. However, the GSH concentration were positively correlated with the concentration of original or weathered PE MPs, means it involved in Cd induced oxidative stress. However, since the original PE MPs and weathered PE MPs have no significant difference in the effects of GSH concentration in plant tissues, GSH may not be the main factor affecting the differences in plant growth and oxidative stress between the original and weathered PE MPs treatments. Obviously, the higher concentration of original or weathered PE MPs application, the greater the damage of Cd to the sweet potato. However, there were no paper has reported about the effect of HMs on MP accumulation in plant tissues.

In our study, Cd increased the PE MPs accumulation in the cortex of roots (**Figures 6E–N**), and reduced PE MPs accumulation in shoots (**Figures 6F–O**). This may be because, in extreme environments or under nutrient-deficient conditions, the transporters of nutrients with poor selectivity than those that operate at the normal nutrient conditions, and when transporting nutrients, they can also transport HMs such as Cd into the plant (Thomas, 2021). As a result, in extreme environments more nutrient elements and Cd adsorbed on the MPs and more MPs were absorbed into the roots. Plants can tolerate HMs at low levels by sequestering it safely into vacuoles in their cells. It is an important mechanism for plants to detoxify these toxic elements, and it is also a key step to limit their transport to the edible parts of crops (Zhao et al., 2022). Therefore, although the accumulation of Cd in roots, stems and leaves of sweet potato under the co-treatment of Cd and PE MPs was higher than that under the single treatment of Cd, the transport factor of Cd was reduced, thus reducing the accumulation of PE MPs in the shoot (**Figure 6**). On the other hand, this may be because, the combined effect of HMs and MPs tends to change from an additive effect in root to an antagonistic effect in shoot (Yuan et al., 2000). In addition, the coexistence of Cd and PE MPs aggravated oxidative stress and inhibited root activity, and also reduce the transport factor of Cd and MP, affecting the absorption of MPs by plants (Kim et al., 2019; Dong et al., 2020; Wang et al., 2020).

## 5 Conclusion

The 5% of weathered PE MPs can significantly increase soil EC compared with control (0% PE MPs) and 5% of original PE MPs. The original PE MPs can significantly adsorb more Cd from solution, and increase Cd accumulation in sweet potato tissues, which lead to higher membrane lipid peroxidation damage in plants and lower biomass compared with weathered PE MPs treatment. No PE MPs were found in the leaves, and higher concentration of PE MPs treatment make higher PE MPs accumulation in roots and stems. Cd increases the PE MPs accumulation in cortex of roots but reduce PE MPs accumulation in shoots. The coexistence of original PE MPs and Cd poses a greater edible safety risk of sweet potato.

## Data availability statement

The raw data supporting the conclusions of this article will be made available by the authors, without undue reservation.

## Author contributions

LS conceived the project, involved in planting of sweet potato and wrote paper. ZC involved in planting of sweet potato and analyzed the data. YH and JL carried out oxygen measurements and enzymatic assays. ZS wrote, reviewed and edited the paper. YC is responsible for conceptualization, funding acquisition, review and editing. All authors contributed to the article and approved the submitted version.
